# Dental Chair

**Published:** 1852-01

**Authors:** 


Dental Chair.—We have recently examined an improvement in a dental chair. The improvement is the invention of Dr. Biddle of Lafayette, Ia., recently of Pittsburgh, Pa. It consists in hinge joints for the arms, so that in letting the back of the chair down, the arms accommodate themselves to the change of position. These hinge joints are made of brass, and are in size the same as the arm pieces to which they are attached. But the most important improvement is the fixture for letting down the back. This consists in a screw fastened to the seat of the chair, with an upright piece extending up, to which the back of the chair is fastened. This is made of cast iron, and is very strong and heavy. The screw is worked by an iron rod running up at the back of the chair. This is out of the way of the operator, and yet just at hand to make such alteration in the change of position as is desired for the patient. The head-rest is the same as that now in general use, and called with us the Philadelphia pattern. This is moved in all directions. The seat of the chair is raised by the same means as the Philadelphia chair, only that the spring which is used in the latter is thrown aside for a far more neat and substantial plan for holding the seat to the position desired; this is a notch wheel and drop. We intended giving proper drawings of the improvements, but could not get them ready in time. Dr. Biddle has patterns for the brass joints and the castings for the back, which he kindly offers to some individual who wishes to manufacture a few of these chairs for sale, without any other cost than that of transportation. We think chairs of this plan can be made for $50, the wood work being of mahogony and the cushioning of good plush. Dr. Biddle, in getting up these patterns, has had mainly in view a substantial, convenient, and durable operating chair for his own use, and we think he has accomplished his object, and now in professional kindness gives it to the profession.

